# Smee on Electro-Biology

**Published:** 1849-04-01

**Authors:** 


Art. VI.—*Elements of Electro-Biology, or the Voltaic Mechanism of Man; of Electro-Pathology, especially of the Nervous System; and of Electro-Therapeutics.* By Alfred Smee, F.R.S., Surgeon to the Bank of England, to the Central London Ophthalmic Hospital, to the Royal General Dispensary, &c. &c. &c. London: Longman. 1849. pp. 154.

The volume before us treats of the relation of electricity to the vital functions during the maintenance of health—of disordered states arising from electrical disturbances in the body—of the influence of electricity on the animal economy—and of its value for the cure or alleviation of various maladies. A work of this character has long been required by the medical profession, and the reason why this want has not already been supplied is, doubtless, owing to the difficulties which the subject presents, as a good surgeon may not be a good physiologist, a good physiologist may not be a good electrician, or acquainted with the intimate working of that most wonderful engine of modern times—the voltaic battery. These difficulties the author seems to have felt, as he states in the preface that he has been for ten years engaged, at intervals, upon the work, and its very slow advance from year to year has been a constant annoyance to himself. The treatise commences with a definition of life.—

"Life is a condition difficult to define, because it does not denote one constant state in the body to which it appertains, but refers to a series of changes continually occurring. The illustrious Bichât considers it to be "the sum of the functions by which death is resisted," but this, to my mind, is not an intelligible definition. If we regard the state of a living animal, we find that it consists of two parts, a solid and a fluid. Between these two parts changes are continually occurring. Life, therefore, comes under our notice only as an idea which we form of a solid and fluid body in a state of action, and thus may be defined to be—'The idea of the performance of certain specific actions between the parenchyma and blood or fluid of an organized being.'

"From the facts which I have already stated, we perceive that the vital functions are divided into two classes, those of animal life, and those of organic life; and to the idea of both collectively we assign the general term of vitality. Life, then, is one word used to signify a number of changes. It is no independent reality apart from the matter which exhibits these phenomena. Neither is it an imponderable attached to matter. Nor is it an all-pervading ether, or *anima mundi*, filling space, as some philosophers would have us suppose; but in its widest signification it is one word used to designate the combined functions of assimilation, growth, nutrition, excretion, the reception of impressions, the registration of impressions, the combination of impressions, together with the production of force, electricity, light, heat, sound, &c. Such is life, an idea necessarily inferring action, and realising the poet's thought,

"She dreads an instant pause, and lives but while she moves."

Our author then particularly investigates the conditions of animal life, and traces the analogy of a living body to the action of a double voltaic battery :—

"A central parenchyma, a peripheral parenchyma, connected together, and each supplied with bright arterial blood, are necessary to life. It follows, therefore, that bleeding causes death—that the supply of imperfect blood, such as carbonaceous blood, is insufficient to life. Moreover, a destruction of the central parenchyma, by injuring the brain, or of the peripheral, by destroying the body, instantly prevents the manifestations of the functions of animal life. Lastly, a separation between the central and peripheral parts, as in the act of pithing, is attended with the same result.

"When action ensues, a change of matter occurs, which is proved by an increased excretion, and corresponding desire for food. This fact demonstrates that the functions of animal life are obedient to the general physical law, that no force can be generated without some corresponding equivalent change of matter.

"Putting all these facts together, we must seek for some physical apparatus which shall agree with the requisites for the maintenance of organic life. Now a central apparatus, supplied with a peculiar fluid, a peripheral apparatus similarly supplied, the whole connected together to form one universal total, is the apparatus desired, and such an apparatus we have in a double voltaic battery.

$$\left\{ \begin{matrix} Z \text{———} S \\ S \text{———} Z \end{matrix} \right\}$$

"Now, if we abstract the proper exciting fluid from either end, or substitute any other fluid, or destroy the structure either at one end or the other, or divide the connecting portions or wires, the effects proper to the apparatus will not be manifested, and the battery will be destroyed."

The actual presence of the voltaic battery in animal bodies, is then demonstrated by what the author calls the electro-voltaic test :—

"If we take two perfectly polished steel needles, and let them remain in any liquid for a short time, so that they may be under the same circumstances, they will exhibit no indications of electricity. When, however, a voltaic current acts upon the liquid, they immediately evince the properties of a voltaic circuit. The one near the negative pole of the battery will become positive; the other, near the positive pole, will become negative; and it is to this circuit that I shall have frequent occasion to make reference during this treatise, and which I propose to designate by the term of Electro-Voltaic.

"By employing the electro-voltaic circuit, we have a test by which we can ascertain the presence of a current in any hydro-voltaic combination, or supposed combination, even if it be limited to a single cell. Having thus obtained the mechanism for examining animal bodies, the time arrived for applying the test to the living creature. The first animal which was honoured was a black rabbit, into the masseter of which I introduced one sewing needle, whilst the second was placed in the subcutaneous cellular tissue. After leaving them for a few minutes, so that they might be in the same state, they were connected with the galvanometer, without sensible deflection of the needle. After a few moments, the animal not liking its treatment, made an attempt to bite my finger, and the deflection of the galvanometer instantly showed the mechanism of volition. I then gave the creature a piece of wood to bite, upon which it used all its power of mastication, and by catching the oscillation of the needle, a very powerful current was exhibited."

From this experiment he infers the body to consist of one battery, which Mr. Smee terms the peripheral battery, and which is connected by the sensor and motor nerves with the central battery.

"Inasmuch as the two needles are placed respectively in the skin and muscle of the animal, it follows that the current is greatest between those two parts, as the needle must be enclosed in the path of the current to exhibit the phenomena. The periphery, or body, therefore, consists of the muscular substance, forming one pole, the cutaneous tissues the opposite, the serous fluid, which lubricates the parts, being the electrolyte. The whole forms a voltaic battery, which I shall hereafter consider in minute detail, as the Peripheral Battery.

"If we follow the course of the nerves, we find that they are prolonged to the brain, and end in the gray matter, where they again come in contact with a large quantity of blood-vessels. As the two series of nerves are not immediately connected in the brain, it follows, according to the laws of voltaic action, that another battery exists there, which may be termed the Central Battery."

After this general description, the sensations are separately studied in detail under terms derived from the Greek, which the following paragraph will sufficiently explain:—

"Electro-Aisthenics, then, comprises the study of all the various organs of sensation, as that of sight, or Opsaisthenics—of hearing, or Ousaisthenics—of taste, or Gumaisthenics—of smell, Rhinaisthenics—of touch, or Cænaisthenics. After a most attentive consideration of the subject, I feel that biology must admit one other sense—namely, that of bodily feeling, or Somaisthenics. By this sense we obtain a knowledge of ourselves, and of the changes taking place in different parts of our own bodies, without which, I shall hereafter show, that we could have no individuality, no personality, and could not even perhaps prove that our bodies did not belong to another individual.

"For the manifestation of the phenomena of all these senses, two conditions are invariably necessary—the presence of a nervous expanse, and the supply of bright arterial blood to that expanse. The universal co-existence of blood and nerve constitutes the foundation of electro-biology, for we invariably find that blood is useless without nerve—nerve inactive without blood—both being requisite for the production of any of the varied phenomena of animal life."

The consideration of the structure of the eye led to the construction of several voltaic circuits, in which the voltaic current was determined by the action of light. These photo-voltaic circuits he distinguishes into positive and negative: in the former of which the light causes the pole acted upon to become positive; in the latter negative, so that, physically speaking, the presence of a voltaic circuit, which is produced by light, is an ordinary phenomenon, although heretofore not recognised by philosophers. After these experiments, the eye is examined by the electro-voltaic test.

"To apply this test to the eye, one needle should be thrust into the eye of an animal through the choroid coat, and a second into the muscle in the neighbourhood; when, if a sudden transition be made from darkness to strong light, a very slight deflection of the galvanometer declares the presence of a photo-voltaic current.

"There are unquestionably considerable difficulties in the operation, but by careful management and watching the oscillation of the needle, the current may be made decidedly appreciable. In estimating these effects, two tests are applicable: first, the motion of the needle in one direction; and 2ndly, the stopping of the oscillation of the needle when the current is reversed. A very feeble current may be ascertained with certainty by this manœuvre."

Mr. Smee has not actually ascertained the presence of the voltaic circuit in the ear or in the tongue, though he gives theoretical structure of these parts. He has, however, ascertained that a current is generated in the nose, but that the animal has extreme repugnance to the operation.

"We may merely, in a remote and imperfect manner, imitate such a state of things, and form an artificial ear, by fixing a piece of vellum over a glass vessel shaped like a funnel, and terminating in an imperfect syphon. When the vellum is thrown into action, the water would be displaced in the tube, and as a consequence therefore, a circuit might be made or broken. By labour, I have no doubt but that a perfect acoustic telegraph could be made, which shall be acted upon by sounds, and have the power of transmitting them to any distance.

"We may make a voltaic battery, in which the circuit shall be determined by savours, in very different methods. For instance, if we place a little per-salt of iron, with two platina poles, in a V-shaped tube, and then drop a little infusion of flesh meat into one side, a voltaic circuit would instantly be produced. In nature, taste is probably excited by the absorption or contact of savours."

The determination of the presence of the voltaic current in common sensation or cænaisthenics, is spoken of as a condition very easily ascertained.

"The determination of the course of the voltaic current is extremely easy, if the cænaisthesis is the subject of experiment. We have only to introduce one needle into the muscular tissue, and a second under the cutaneous structure, when a distinct current is immediately manifested in the galvanometer, when the animal is pinched or otherwise irritated. From this experiment we learn that the cænaisthetic pole is positive, the muscle negative. Of course the electro-voltaic current, by which we render manifest this phenomenon, is in the reverse direction."

Mr. Smee states that feeling may be excited by either heat or force, and that voltaic circuits, determined by variations of temperature, may be easily constructed. These he terms thermo-voltaic circuits; and with regard to force, the following curious illustration is given:—

"There is no difficulty in the consideration of a voltaic circuit excited by force, for if by pressure we prevent the arterial corpuscule from coming in contact with the nerve-fibre, action must arise, inasmuch as the balance would be destroyed; polarity would ensue, and action would take place.

"We may imitate this kind of circuit by very easy means. For instance, if we take two pieces of iron wire, and insert them in very dilute acid, no action ensues when tested by the galvanometer. The same result is obtained if the blood corpuscule be imitated by taking a membrane containing a little nitrate of iron, and placing one such artificial corpuscule against such iron wire. As soon, however, as the artificial corpuscule is thrust aside from the pole, a very powerful current is generated, which has its origin at that pole where no corpuscule exists. This structure is strictly and perfectly analogous to the natural mechanism of the body."

Up to this point the subject is quite within the range of experiment, the mechanism of sensation is determined, and the sensations are carried by the nerves or bio-telegraphs to the brain, when they end in the gray matter in contact with minute arterial trunks. Mr. Smee gives the following process for injecting this organ:—

"I have been enabled to make the most beautiful injections of the brain and spinal marrow which have ever been executed, by using an injection consisting of carmine dissolved in ammonia, and mixed with a solution of isinglass. This injection is of an intense colour, perfectly fluid, and is thus enabled to penetrate the minutest ramifications of the capillary vessels. In injecting the brain, it is necessary to use one that is perfectly fresh; and I generally inject immediately after the animal is killed. By these means the most exquisite injections of the brain and spinal cord have been executed; and in all my preparations, it is shown that wherever gray matter exists the blood is distributed, and where the white matter exists there is no blood; and from these considerations, physiologists infer that the gray matter is the active part of the brain."

The sensations when they have acted upon the brain are remembered.

"When a man receives an impression it is not evanescent, passing immediately away, but it is retained in the system to regulate future actions. Now, in voltaic constructions, it is not difficult to produce an action which shall influence future motions, and thus exhibit the effects of memory."

For the structure of the brain itself, Mr. Smee considers that the following is the mode in which the fibres may be arranged. Units of sensation are received in the first battery. These acting in a second, show what combination of sensations are appreciated at one time. By the connexion of all these into one set of fibres for each sense in the third battery, we distinguish the sense by which the impression was received, as whether it was received by vision, hearing, smelling, or other sense. When these are associated together, we learn the combined properties of bodies—that is, whether the impression is derived from the action of two or more senses at one time; and, lastly, he supposes that the fibres are connected together into one total pole. He observes—

"The position of this battery is somewhere in the centre of the Pons Varolii; and when I have plunged a needle into that situation on one or two occasions, I believed that I have observed deflection in the galvanometer. The animal has been, however, invariably killed; and therefore I have not been able to repeat this experiment so often as I could otherwise desire, nor could I judge of the result in so satisfactory a manner as to pronounce positively upon it.

"This completes the structure of the brain, as inferred from voltaic laws; and it is now manifest, that every structure here assumed may be imitated and repeated by voltaic combinations."

Desire to act is considered to be a state of tension, and the laws regulating action are thus expressed—

"It is manifest, by this arrangement, that any circumstance determining the action of the fibres of the aisthenic battery would, or might influence the whole upon certain laws.

1. Each sensor or aisthenic nerve is opposed to every motor or dynamic nerve, and may thus excite it to action.
2. This circuit would be completed through the nearest motor nerve of the body, because that would be the readiest course, unless there were obstacles offered in some part of that circuit, or adjuvants added to other parts.

3. If any obstacles were offered in any part of the course, the circuit would be completed by some other motor nerve, according to the facility with which the current could pass."

From the five central batteries, either separately or combined, the author deduces the properties of the mind, and states his belief that by them we are enabled to estimate the time of the occurrence and cause of an event, as well as the form, size, magnitude, and number of objects. He considers them to be the source of consciousness, and other mental phenomena. Mr. Smee thus writes—

"The mind, however, derives certain powers from combinations of two or three of these batteries conjointly. We obtain the ideas of a thought and of a reality in this manner. An idea is a thought when the bodily action does not concur with the combination which appears to the mind. An idea is a reality if these two combinations do concur. There are times, when indulging in the spontaneous thoughts of the mind, that a question arises to ourselves, whether everything around us is not a dream—a fanciful creation of the mind; and in such a state, we are led to doubt whether it be possible to prove our very existence; but the moment we ascertain whether actions of thought concur with actions in the body, the difficulty ceases, and we are enabled to distinguish immediately between a reality and a fanciful creation of the brain. This power is termed consciousness.

"Other combinations, doubtless, give us other ideas; thus personality and infinity give us the idea of the soul; pleasure and infinity, of good; pain and infinity, of bad; cause and infinity, of God; time and infinity, of eternity; infinity, pleasure, and time, of Heaven; infinity, pain, and time, of hell. Personality and all the units of sensation give us the idea of the body; personality, infinity, and time, of immortality. Personality and other totalities of senses, give us the idea of the mind; thought and infinity, of spirit. Lastly, action, infinity, and pleasure conjoined, give us the idea of virtue; action, infinity, and pain, of vice."

Having traced the sensor nerves to the brain, the author traces the dynamic nerves to the muscles, electrical organs, &c. He says—

"Each muscular fibril is completely enveloped by blood-vessels, which run parallel to the fibrils. The supply of bright arterial blood is absolutely necessary for the manifestation of muscular motion, and, consequently, the relation of the capillaries should be fully borne in mind. A good mode of injecting the capillaries is by the exquisite carmine injection before described, when treating of the mode of injecting the textures of the brain.

"According to this view of the case, muscular contraction ensues from the material existing in the ultimate fibre being increased in bulk by changes taking place in consequence of the voltaic circuit.

"An artificial muscle may readily be constructed, to act upon a similar principle. To effect this object, the sheath of the ultimate fibre should be imitated by a bladder, or perhaps more strictly by a piece of the gut of any animal. Into the interior of this, a strip of platinized silver and a small quantity of sulphuric acid should be introduced. The gut or bladder should be tied round at both extremities by a piece of strong cord, which would close the apertures, and serve for artificial tendons. The whole must then be immersed in dilute sulphuric acid, containing a positive pole of zinc. When the zinc and silver are connected, gas is evolved within the artificial muscular fibre, when it widens and shortens. This contraction acts upon the artificial tendons, to produce any required motive power.

"An arrangement similar to that of the battery of the electric fish may be made in various ways. The arrangement, which accords most nearly with nature, I shall term the artificial electrical fish. This artificial electrical fish is made by taking an ordinary solution of ferrocyanate of potash contained in a glass vessel. Into this glass vessel, a porous cell with a similar solution is introduced. Now, if a series of these cells be taken, and connected together by platinum wires, so arranged that the inside of the porous cell of one vessel be connected with the interior of the second by a platinum wire, no action will be indicated by the galvanometer. If, however, a current of voltaic electricity be now passed through each cell from the porous tube to the exterior, one compartment, or the hydrogen side, will become alkaline, and the salt will retain its chemical character; the other cell will become acid, and be converted into the red prussiate."

We have not time to follow the electrical relations of sleep and rest, nor can we enter into the consideration of the changes taking place during electrolyses.

"To charge," says Mr. Smee, "the electro-biological batteries, we must take suitable food; and to keep them in working order, we must eliminate the changed material. The food we take is changed into blood; and electro-biology shews that the blood is the vivifying agent, and explains how the blood of any animal may, in any sense, be said to be '*the life thereof*.'"

The relation of electricity to cell-life is not so obvious as in animal life. It appears to have remarkable effect on the circulation of the blood. The author remarks:

"With regard to the cells of animal bodies, one of the most wonderful and extraordinary results which I have observed is the action of electricity derived from the intermittent current of the various forms of electro-magnetic machines. When a frog's foot is arranged in the field of the microscope, and the intermittent current is directed through the animal, the circulation instantly stops, as though by magic. The current in the veins, indeed, seems slightly to retrograde, though it still continues its course for a short period in the arteries; the whole effect giving the appearance of all the corpuscules having a tendency to be drawn into the capillaries."

Our author quotes the experiments of Cross and Weekes, and gives some illustrations of the acari, which they suppose to have obtained from the action of electricity. Mr. Smee states, that Mr. Weekes has lately observed two more species produced from other solutions. The author says :

"It becomes now a matter for investigation, how far a totally different organic being may spring from another organic body made up of cells. In practice, we find that animals are found in internal parts of other animals, where by no possibility they could have been carried, either in the form of an egg, or of a living creature. We find, also, that from organic matter, other organic forms are continually arising, without proof of any seed or germ having been placed there. From these considerations, we are led to inquire, whether external forces may so act upon the cell as to give rise to a totally different form of organization. It still remains, however, an unsolved question whether parasitic fungi, as that found in the ringworm of man, parasitic creatures, as the *echinococcus hominis*, the tapeworms, and other bodies are produced by virtue of the cells of the human being taking on new forces, and aggregating in new directions; or whether the germs are carried there in some unknown method."

In a subsequent chapter, the curative influence of electricity is considered in all its forms as frictional electricity, lightning electricity, animal electricity, hydro-electricity, thermo-electricity, coil and electro-magnetic electricity, and magneto electricity. Of these instruments, he states:

"For electro-therapeutics, or even for all purposes, it is advisable that the current should act in one uniform direction, and that it should not be a to-and-fro current, as ordinarily produced. Whenever an instrument is bought, this fact should be ascertained, which may be readily effected by finding whether metals are reduced from solutions of their salts, on only one of the platinum poles attached to the terminations of the wires.

"The magneto-electric apparatus possesses an advantage in its not requiring the aid of a voltaic battery to set it in action. It is always ready, without any preparation, for the purposes of the analyst or therapeutist, in all weathers and at all times. Its only disadvantage is a trifling additional cost in the first instance, in consequence of the price of the permanent magnets, and it moreover requires a certain amount to keep its armature revolving."

Mr. Smee further considers the influence of magnetism upon the body, of heat derived from electricity, of light derived from the same source, and of its power to effect decomposition. He says:

"Voltaic electricity decomposes all binary compounds. It resolves water into its elements, oxygen and hydrogen; and salts into their acids and bases. Various propositions have been made to use this property for the cure of disease; and I have seen recommendations to employ it for cataract, by inserting one needle into one part of the lens, and a second into another. Nothing but the most frightful ignorance could have dictated such a recommendation. I tried the experiment upon the eye of a perfectly healthy rabbit; the poor beast appeared to suffer the most excruciating agony. The ball of the eye was distended with gas on the application of the current; the cornea, in a few minutes, became quite opaque, and the whole eye was finally destroyed."

Lastly, the effect of temperature and light, in its power of exciting the voltaic actions of the body, are studied, and also the effect of physical and mental impressions on the circuit are discussed.

The last chapter is devoted to the electrical relations of various diseases, and the benefit of electricity in each separate malady is described. The author concludes with the following paragraph:

"In submitting this work to the public, I may state that its development has afforded to me unmixed delight. And with respect to the opinion which other philosophers, after due deliberation, may be led to form of its contents, I can only say, in the words of the immortal Harvey: 'Spes mea in amore veritatis et doctorum animorum candore.'"

The above is a short analysis of this, Mr. Smee's valuable and interesting volume, though, from the whole being so interwoven, each part being dependent upon the antecedent, the work very ill bears this attempt to extract particular portions. We have reason to believe that Mr. Smee's researches in electro-biology have attracted the notice of some of the most learned *savans* of the day. He is entitled to great credit for the persevering devotion with which he has, almost unaided, pursued his scientific experiments. His work does him infinite credit.

